# Supplementary material for: Effect of Functionalization of Texturized Polypropylene Surface by Silanization and HBII-RGD Attachment on Response of Primary Abdominal and Vaginal Fibroblasts
Source: Polymers (Basel). 2024 Feb 29;16(5):667. doi: 10.3390/polym16050667 (PMC10934969; doi:10.3390/polym16050667)
Supplement: Supplementary file 1 [file polymers-16-00667-s001.zip › polymers-2851244-supplementary.pdf]

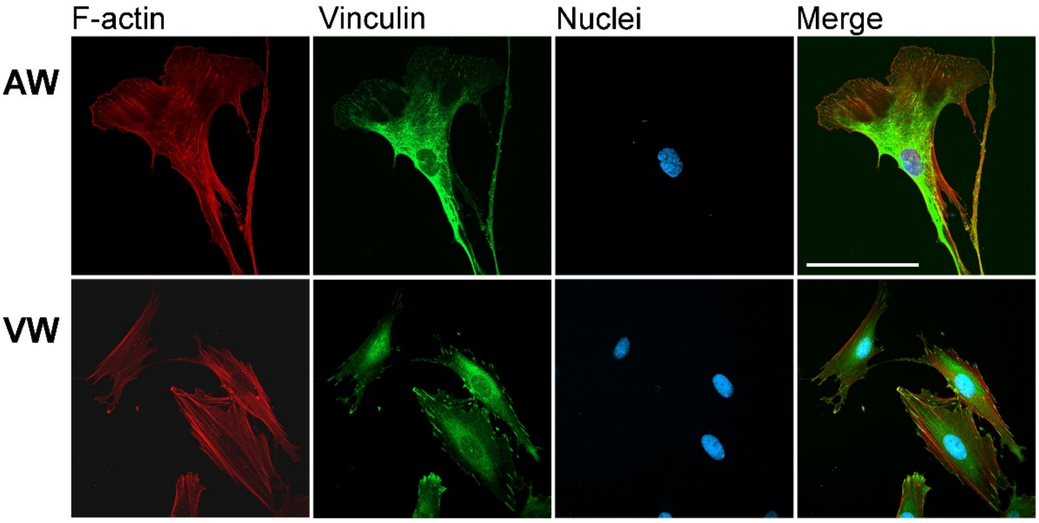

Supplementary Figure S1. Filamentous actin (F-actin) and vinculin staining of primary abdominal (AW) and vaginal wall (VW) fibroblasts. Cells were seeded onto 18-mm glass coverslips in 12-well plates for 24 hours, fixed for fluerescent staining and visualized by confocal laser scanning microscopy. Actin filaments were tagged with rhodamine labeled phalloidin (red), vinculin was labeled with mouse anti-vinculin antibody and the corresponding Alexa Fluor 488-conjugated secondary anti-mouse IgG1 antibody (green), and nuclei were stained with DAPI (blue). Scale bar represents 50 μm.
